# Messing with Signal 1: How Perturbed MHC Class I Antigen Presentation Contributes to Cancer

**DOI:** 10.3390/cells15070653

**Published:** 2026-04-07

**Authors:** Myriam Lawand, Salman Al Rayess, Rawad Jaber, Peter van Endert

**Affiliations:** 1 Department of Biology, Faculty of Arts and Sciences, Souk El-Gharb Campus, University of Balamand, Aley 1501, Lebanon; 2 Université Paris Cité, Institut national de la santé et de la recherche médicale (INSERM), Centre national de la recherche scientifique (CNRS), Institut Necker-Enfants Malades, 160 rue de Vaugirard, F-75015 Paris, France; 3 Service Immunologie Biologique, Assistance Publique-Hôpitaux de Paris (AP-HP), Hôpital Universitaire Necker-Enfants Malades, 149 rue de Sèvres, F-75015 Paris, France

**Keywords:** antigen presentation, antigen-presenting cell, MHC class I, T cells, cytotoxicity, cytokine, cancer

## Abstract

The antigen presentation machinery processes proteins for presentation to T cells, thereby controlling activation of the adaptive cellular immune response. Perturbation of this machinery has been linked to the development of various diseases. This review describes the function of the Major Histocompatibility Complex class I antigen presentation machinery and highlights how its perturbation can lead to compromised immune function and disease progression in the context of cancer. We categorize these perturbations into four distinct mechanistic levels: peptide generation, peptide loading, MHC class I integrity, and epigenetic regulation. This enables an integrated view of their functional impact on immune recognition, supporting therapeutic efforts to target antigen presentation or exploit these alterations in cancer.

## 1. Introduction

T lymphocytes recognize antigenic peptides presented in the context of Major Histocompatibility Complex (MHC) molecules. Naïve T cells require antigen presentation by professional Antigen-Presenting Cells (APCs) for activation, whereas previously activated or memory T cells can be stimulated by antigens presented by a broader range of cell types. Recognition of peptide-MHC complexes by the T-cell receptor (TCR) constitutes signal 1, the primary antigen-specific activation signal, which is necessary but not sufficient for mounting a productive T-cell response.

Professional APCs are key players in activating the adaptive immune response, mediating the coordination between innate and adaptive immunity. They are activated upon recognition of Pathogen-Associated Molecular Patterns (PAMPs) or Danger-Associated Molecular Patterns (DAMPs) through innate immunity receptors. APCs, stimulated by these danger signals within inflammatory tissue environments, capture antigens, process them intracellularly, and subsequently migrate to secondary lymphoid organs (SLOs) to activate naive T lymphocytes [1]. This activation requires the presentation of antigenic peptides on MHC class I and II molecules. MHC class I molecules present to CD8+ T cells, whereas MHC class II molecules present to CD4+ T cells. This process links innate immune activation to antigen-specific adaptive responses. Activated CD8+ T lymphocytes then travel from their site of activation in SLOs to peripheral tissues where they target tumor cells displaying the specific antigenic peptides on their self-MHC class I molecules. Hence, tumors that disrupt any step of the MHC class I antigen presentation pathway gain a survival advantage, explaining why signal 1 perturbations are among the most frequent mechanisms of immune escape across cancer types. Consequently, this pathway is particularly relevant for understanding tumor immunogenicity and improving immunotherapeutic strategies.

Our review provides an up-to-date overview of MHC class I antigen presentation machinery (APM), which is central to CD8+ T-cell-mediated immune surveillance and a key mechanism for cytotoxic lymphocyte recognition of malignant cells. We describe the principal components of the MHC class I APM, examine their perturbations in cancer, and discuss how these alterations shape immune responses and influence disease outcomes.

While numerous reviews have used an integrated conceptual framework to address MHC class I antigen presentation machinery (APM), these studies differ in scope and conceptual focus. As a result, they do not fully capture how distinct APM alterations converge functionally. Several studies position antigen presentation defects within the broader context of immune evasion and epigenetic regulation, integrating APM changes alongside multiple immunoregulatory mechanisms. Although informative, this perspective often limits mechanistic resolution at the level of individual APM components and their direct functional consequences [2]. Conversely, other reviews provide detailed analyses of selected components, particularly MHC class I molecules and β2-microglobulin, with emphasis on immunoediting and therapeutic applications such as neoantigen vaccines. However, this focused approach does not comprehensively address the full spectrum of APM components and their coordinated contribution to antigen presentation [3]. In parallel, some frameworks categorize APM alterations according to hierarchical levels of biological regulation, spanning genetic to post-translational processes, as exemplified by Dhatchinamoorthy et al. (2021) [4]. While valuable for understanding regulatory control, such classifications describe upstream mechanisms and do not explicitly resolve how these alterations translate into functional defects within the antigen presentation pathway.

To address these limitations, our review adopts a function-centered framework that classifies APM perturbations into four mechanistic layers: peptide generation, peptide loading, MHC class I integrity, and epigenetic regulation. This classification enables a clearer link between molecular alterations and their functional consequences in antigen presentation. This allows the identification of alteration types that are most actionable for therapy and predictive of immunotherapy response, providing a unique structural perspective. Therefore, by organizing APM disruptions mechanistically, this review highlights those with potential clinical relevance and provides a foundation for designing strategies to restore or exploit antigen presentation in cancer.

To provide a rigorous and comprehensive synthesis of the field, this review is based on a targeted literature search. Relevant studies were identified using keywords related to MHC class I, APM components, tumor immune evasion, and immunotherapy. Both original research articles and high-quality reviews were considered, with selection guided by conceptual relevance, mechanistic insight, and their contribution to understanding how APM alterations shape antigen presentation and influence immune recognition. This synthesis integrates findings from molecular, cellular, and translational studies, highlighting APM function, dysregulation, and implications for therapeutic strategies. While not a systematic review, this approach was designed to capture the most significant mechanistic and translational developments in the field, emphasizing the functional consequences of APM perturbations and their relevance to tumor immunity and immunotherapy outcomes.

## 2. Key Mechanisms of Antigen Presentation in Immune Surveillance and Diseases

### 2.1. Pathways of Antigen Processing for Presentation by MHC Class I Molecules

Antigens are loaded as peptides onto MHC molecules expressed by APCs. The peptide-MHC (pMHC) complexes are transported to the cell surface for subsequent presentation to T lymphocytes. In humans, MHC molecules are also referred to as Human Leukocyte Antigens (HLAs). MHC molecules are transmembrane glycoproteins encoded by genes located on the short arm of human chromosome 6 [5]. Genetic MHC polymorphism enables T cells to mount specific responses to a wide array of antigenic peptides.

In this section, we describe antigen presentation pathways with a focus on MHC class I molecules, which play a central role in tumor immune surveillance. These molecules present antigens to cytotoxic T lymphocytes (CTLs), which recognize peptide-MHC complexes via their TCRs. While CD8+ CTLs directly mediate tumor cell killing, CD4+ helper T cells, activated through MHC class II antigen presentation, provide essential cytokine support and co-stimulatory signals that enhance CTL priming. This interaction enables activated CTLs to kill malignant cells presenting tumor-derived peptides.

MHC class I molecules are involved in presenting both endogenous (derived from proteins synthesized in the presenting cell) and exogenous (derived from proteins internalized by it) peptide antigens to CD8+ T cells. The latter can be produced in different pathways, often referred to as cytosolic and vacuolar.

#### 2.1.1. Overview of the Endogenous MHC Class I Antigen Presentation Pathway

Endogenous antigen presentation, operating in all nucleated cells, mediates the display of peptides synthesized within the APC to cytotoxic CD8+ T cells in a pathway involving the Transporter associated with Antigen Processing (TAP) and the proteasome, a large cytosolic multi-catalytic protease. Peptides presented through the endogenous pathway originate from cellular proteins including tumor proteins upregulated due to malignant transformation, encoded in the cellular genome [5]. Both misfolded or improperly assembled proteins, and proteins at the end of their functional life cycle are known to be recruited for production of MHC class I presented peptides. MHC class I ligands can also derive from microbial proteins in case of infection by viruses exploiting the cellular translation machinery or even by bacteria invading the cytosol or leaky cellular organelles.

The term “processing” refers to multiple steps preceding peptide loading on MHC molecules. Ubiquitination is frequently (but not always) the first step for protein degradation by a cytosolic multi-enzymatic complex, the proteasome. Degradation of many proteins by the proteasome operates through an ATP- and ubiquitin-dependent mechanism that breaks proteins down to short peptides, typically ranging from 3 to 22 amino acids. These peptides can be further processed into single amino acids that are recycled for synthesis of new proteins. A variant of the proteasome, known as the immunoproteasome, plays an important role in immune responses. It is induced in response to inflammatory signals, such as interferon-gamma (IFN-γ), and predominantly found in immune cells, including human T cells, B cells, and professional APCs [6]. The immunoproteasome differs from the standard proteasome by replacement of all three “constitutive” catalytic subunits: β1 (PSMB6), β2 (PSMB7), and β5 (PSMB5) with three IFN-γ inducible subunits: β1i (PSMB9 or LMP2), β2i (PSMB10 or LMP10 or MECL-1), and β5i (PSMB8 or LMP7). The constitutive subunits are expressed across all cell types, maintaining baseline protein turnover for cellular functions like metabolism and cell cycle regulation. In contrast, immunoproteasomes generate peptides better suited for MHC class I presentation [7], thereby improving CD8+ T-cell recognition of infected or malignant cells to elicit effective immune responses.

Proteasome-mediated degradation results in the generation of peptides of variable length [8]. A fraction of these peptides is transported by the heterodimeric peptide transporter (TAP), composed of the TAP1 and TAP2 subunits, into the endoplasmic reticulum (ER) [9].

During their formation, MHC class I heavy chains and β2-microglobulin (B2M) molecules associate with chaperone proteins residing in the ER (calnexin (CANX), calreticulin (CRT), tapasin (also known as TAPBP: TAP-binding protein) and ERp57 (PDIA3: protein disulfide isomerase family A member 3). Together with the TAP transporter, these chaperones form the peptide-loading complex (PLC). Peptides exceeding the optimal length of 9–10 amino acids require further processing. These peptides undergo N-terminal trimming by ER aminopeptidases (ERAP1 and ERAP2). Peptides of 8 to 10 amino acids are finally loaded onto MHC class I molecules. Binding of optimally adapted peptides stabilizes MHC class I molecules, which then dissociate from the PLC and are transported to the Golgi compartment and then to the cell surface to present peptides to CD8+ T cells.

#### 2.1.2. MHC Class I Cross-Presentation Pathways

MHC class I molecules are also capable of presenting peptides derived from exogenous proteins internalized by phagocytosis, macropinocytosis, or endocytosis to initiate cytotoxic CD8+ T cell responses. This process, termed “cross-presentation” [10,11], holds an important role in various processes such as the induction of immunity against viruses that do not directly infect APCs, or against tumors that are not of hematopoietic origin [12,13]. Cross-presentation is also required to induce immune tolerance [14].

The TAP transporter and the proteasome are thought to play an important role in the “cytosolic” pathway of cross-presentation. Pharmacological inhibition of the proteasome was shown to inhibit the presentation of exogenous antigens on MHC class I molecules. The effects of proteasome inhibition are difficult to interpret because of the enzyme’s multiple roles in cellular physiology. Nevertheless, these observations suggest that cross-presentation may involve the transfer of antigens from endo/phagosomes into the cytosol for degradation [15]. Subsequent studies indicated a role of TAP in this pathway; this may indicate transport of cross-presented peptides from the cytosol into intracellular compartments such as the ER, phagosomes, and endosomes and alternatively highlight the importance of recycling cell surface MHC class I molecules for cross-presentation (TAP-deficient cells have very low levels of cell surface MHC class I molecules) [16,17,18,19,20,21,22,23].

Unlike the cytosolic pathway, the so-called vacuolar pathway of cross-presentation operates independently of the proteasome, as antigen presentation remains intact even when the proteasome is inhibited [24]. A study examining the role of TAP-L (TAP-like), a homodimeric lysosomal peptide transporter, in cross-presentation demonstrated its significant recruitment to dendritic cell (DC) phagosomes at a late stage, posterior to antigen degradation [25]. TAP-L may play a role in phagosome maturation; its deficiency leads to accelerated maturation characterized by increased recruitment of late lysosomal markers and enhanced degradation of phagocytosed antigens and in vitro transported peptides [25]. These findings suggest that TAP-L may regulate antigen-containing compartments, indicating its potential involvement in the vacuolar pathway of cross-presentation.

### 2.2. Antigen Presentation, Tumor Immunogenicity and Immune Evasion in Cancer

Impaired MHC class I antigen presentation is a common mechanism by which cancers evade immune detection and represents a significant obstacle to effective anti-tumor immunotherapy.

Multiple mechanisms contribute to the regulation of antigen processing and presentation, ultimately shaping tumor immunogenicity, defined as the capacity of tumor cells to express antigens capable of eliciting an immune response. These antigens are either uniquely tumor-specific or overexpressed relative to normal tissues. These antigens are presented in a manner that activates immunity rather than inducing tolerance. This is influenced by the interplay between the tumor’s inherent antigenicity, its ability to process and present antigens, and the immunosuppressive factors in the tumor microenvironment (TME) [26]. It is important to distinguish between tumor antigenicity (the presence and abundance of potentially recognizable antigens), antigen presentation competence (the functionality of MHC and the APM), and immunosuppressive mechanisms within the TME, each of which can independently influence the capacity of T cells to recognize and respond to tumor antigens. These factors represent distinct but interconnected dimensions of tumor-immune interactions. A compromised immune response can contribute to an immunosuppressive TME, further enhancing cancer progression [27].

In this context, tumor antigens can be categorized into tumor-associated antigens (TAAs) and tumor-specific antigens (TSAs) or neoantigens [28]. TAAs are normal proteins produced by unmutated genes that are often overexpressed in tumor cells relative to normal cells. TAAs often induce tolerance, which however can be overcome by their over-expression in tumors. In contrast, neoantigens arise from somatic mutations in cancer cells, leading to the creation of unique peptides absent in normal tissues and therefore identified as foreign by the immune system [29]. While targeting TAAs may result in autoimmune toxicity due to their expression in healthy tissues, neoantigens are perceived as non-self by the immune system and thus evade tolerance mechanisms [28]. The presentation of neoantigens is crucial for stimulating robust anti-cancer immune responses [30]. However, the presence of few neoantigens alone is neither necessary nor sufficient to predict response to immunotherapy. While many tumors express few neoantigens and may therefore be less responsive to immunotherapy [31], clinical outcomes also depend on the integrity of antigen presentation pathways and the degree of immune suppression in the TME. Under these conditions, tumors with low neoantigen burden can still respond, whereas tumors with high neoantigen load may remain unresponsive if APM function or the immune contexture is compromised.

Immune evasion is a fundamental feature of tumor progression, whereby cancer cells escape immune destruction through multiple mechanisms, including immunoediting [32], HLA downregulation [33], secretion of immunosuppressive mediators [34], and expression of immune checkpoint (ICP) ligands.

Immunoediting is a dynamic process in which immune pressure shapes tumor evolution by selecting tumor variants with reduced immunogenic antigen expression, while additional immune escape mechanisms may arise from alterations in the TME. This process is divided into three phases: elimination, where the immune system detects and destroys cells expressing antigens efficiently identified by it; equilibrium, where low levels of cells expressing other tumor antigens persist but their proliferation and dissemination are held in check by the innate and adaptive immune responses; and escape, where additional mutations in the surviving tumor cells allow for proliferation and tumor growth.

Other factors within the TME, including ICP-signaling pathways, can regulate lymphocyte effector function and promote immune tolerance to tumor cells. Cancer cells and infiltrating APCs express inhibitory ligands that interact with effector T cells and inhibit their effector function. Regulatory T cells (Tregs), macrophages, and certain DC subsets can provide inhibitory signals or secrete inhibitory cytokines that counteract tumor immunogenicity and promote tumor progression [26]. For instance, interleukin-10 (IL-10) plays a significant role in immune regulation, suppressing antigen presentation and T cell activation, a phenomenon documented across various cancer studies [35,36,37,38,39]. IL-10 has been shown to downregulate the expression of costimulatory molecules, such as CD86, and MHC class II proteins on APCs, including macrophages and DCs. This downregulation can induce anergy in CD8+ T cells recognizing tumor antigens, suppressing their cytotoxic function. Furthermore, IL-10 inhibits the production of pro-inflammatory cytokines, including IL-12, by macrophages. Given that IL-12 is essential for mounting effective anti-tumor T cell responses, its inhibition by IL-10 further promotes immune suppression within the TME. Clinically, elevated serum IL-10 levels in cancer patients frequently correlate with poorer recovery outcomes in both solid and hematological malignancies, highlighting its potential as a prognostic marker [40].

The downregulation of MHC class I molecules is a major mechanism of immune evasion, as cell surface presentation of tumor-derived antigens by MHC class I is essential for activating CD8+ T cell-mediated adaptive anti-tumor immune responses. Tumors evade immune detection by reducing antigen visibility to CD8+ T cells through multiple mechanisms, including genetic and epigenetic alterations that impair the availability of MHC class I molecules for presenting tumor epitopes on the cell surface [2,4].

**Figure 1:**
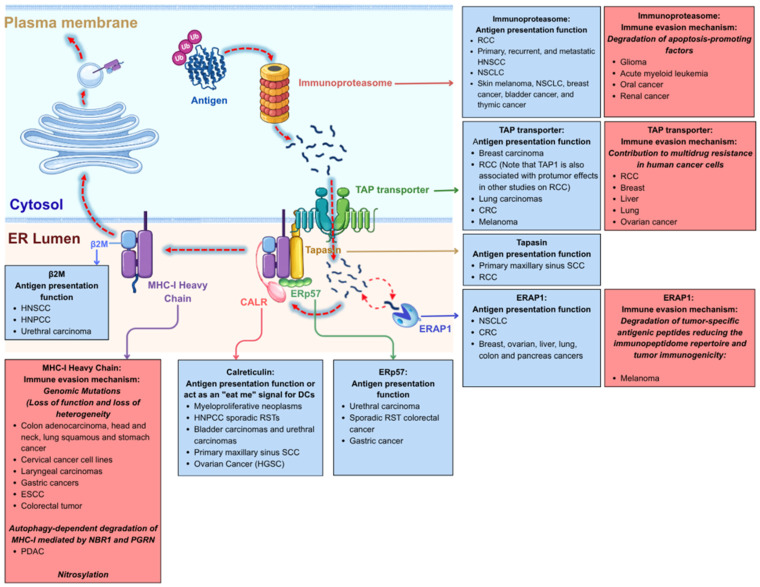
**The MHC class I antigen presentation machinery in cancers: From immune surveillance to immune evasion mechanisms.** Overview of the MHC class I antigen processing and presentation pathway and its dual role in cancer. Under physiological conditions, coordinated activity of the immunoproteasome, TAP transporter, peptide-loading complex, and associated chaperones ensures effective MHC class I presentation of tumor-derived antigens to CD8+ T cells. However, tumors frequently exploit alterations in these components to reduce antigen presentation, thereby promoting immune escape and therapeutic resistance.

Effective antigen presentation depends on the correct function of multiple components of the APM, including proteasomal degradation of antigens, peptide transport into the endoplasmic reticulum, assembly of the PLC, and stable presentation of peptide-MHC class I complexes on the cell surface [41]. Tumors frequently exhibit loss or downregulation of HLA class I expression, enabling immune evasion. The expression levels of the APM genes, including HLA-A, HLA-B, HLA-C, B2M, TAP1, TAP2, TAPBP, ERAP1/2, and immunoproteasome subunits such as PSMB8 and PSMB9, have been correlated with various tumor immune characteristics and patient outcomes. For instance, a higher expression of APM components has been associated with increased infiltration of CD8+ T cells within tumors, reflecting a more robust anti-tumor immune response [42,43]. Conversely, low expression of these genes can correlate with poor prognosis and reduced effectiveness of immunotherapies, such as immune checkpoint inhibitors (ICIs) [44]. In the following sections, we will explore the roles of individual APM components in cancer (Figure 1) and discuss how alterations in APM genes can shape the tumor immune environment and affect the effectiveness of immunotherapeutic strategies.

### 2.3. Key Actors in the MHC Class I Presentation Pathway: Defects and Consequences in Cancer

**Figure 2:**
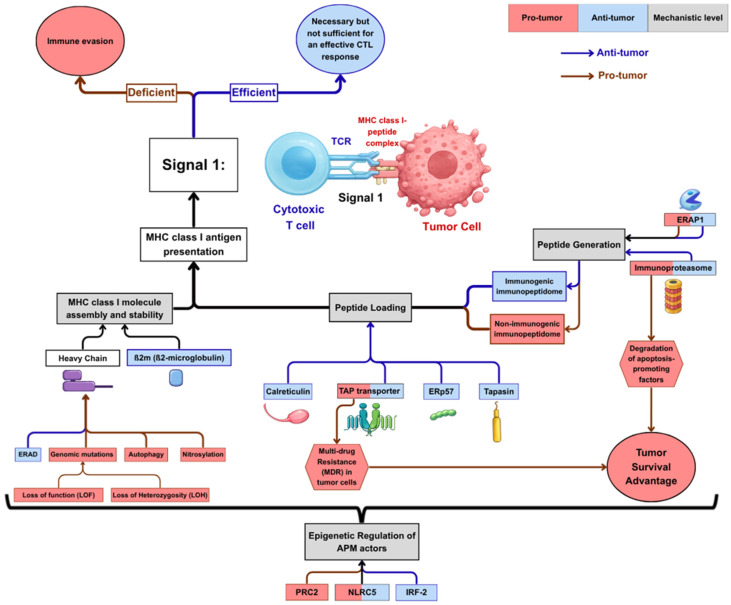
**Integrated MHC Class I APM pathways shaping signal 1 in tumor-immune interactions.** Schematic representation illustrating how major regulatory pathways of the MHC class I APM collectively shape tumor–immune interactions across multiple mechanistic levels: (i) MHC class I stability, assembly, and cell surface expression; (ii) the immunogenicity of the presented peptide repertoire; and (iii) the activation of immune evasion mechanisms. These interconnected processes highlight the integrated control of antigen presentation and its impact on signal 1, thereby determining the efficiency of the cytotoxic T cell response.

Given that antigen presentation is a complex, multistep process involving numerous components, tumors can interfere with this pathway at several levels. To provide a structured framework, APM components can be organized into four groups based on their functional roles in antigen processing and presentation (Figure 2). These include (i) enzymes involved in peptide generation, (ii) the peptide-loading complex, (iii) the MHC class I molecule, and (iv) epigenetic regulators of APM components (Table 1). When evaluating the effects of altered APM components, one should consider that MHC class I levels reflect the amount of cell surface MHC class I molecules expressed, representing the overall antigen presentation capacity. The immunopeptidome describes the quality and diversity of antigens that can be presented on these MHC class I molecules reflecting the tumor’s antigenicity, while the immune infiltrate reflects the biological response downstream by measuring the presence, type and activity of immune cells within the TME.

#### 2.3.1. Peptide Generation: Roles of the Immunoproteasome and ERAP

The initial step in MHC class I antigen presentation is the generation of peptides from intracellular proteins. This occurs through proteasomal degradation by the constitutive proteasome providing general protein turnover across most cell types, including tumor cells, and the immunoproteasome, a variant producing a distinct set of peptides under inflammatory conditions, such as IFN-γ exposure. These differences influence which peptides are more likely to bind MHC class I molecules and be recognized by CD8+ T cells [45,46,47]. Peptides that are initially too long are further processed by ER-resident aminopeptidases, ERAP1 and ERAP2, to achieve the optimal length for stable MHC class I presentation, thereby shaping the repertoire of antigens displayed on the cell surface [48].

##### The Immunoproteasome

Given that immunoproteasomes can influence the repertoire of peptides available for MHC class I presentation, defects in immunoproteasome subunits are frequently associated with impaired peptide generation [49]. A preliminary study involving 54 cases of renal cell carcinoma (RCC) revealed that high-grade tumors exhibited low expression levels of the immunoproteasome subunit genes LMP2, LMP7, and LMP10. Notably, these low expression levels were significantly associated with worse prognosis in RCC patients [50]. These findings suggest that reduced expression of immunoproteasome subunits compromises tumor antigen presentation.

In a subsequent study in HNSCC, immunohistochemistry, immunoblotting, and RT-PCR were used to evaluate protein and transcript levels of LMP2, LMP7, and the peptide transporter TAP1 [51]. This study demonstrated downregulation or loss of these IFN-γ-inducible components in tumor biopsies and cell lines from primary, recurrent, and metastatic HNSCC, which correlated with lower MHC class I levels, poor patient survival, and increased disease recurrence. However, these defects were corrected by incubating the cells with IFN-γ in vitro, suggesting that deficiencies in IFN-γ-inducible APM components may directly impair MHC class I levels.

An integrative genomic and proteomic analysis revealed that non-small-cell lung cancer (NSCLC) cells undergoing epithelial-to-mesenchymal transition (EMT) exhibited reduced expression of immunoproteasome subunits [52]. Another study of 155 NSCLC patients used immunohistochemical analysis and reported high β5i (LMP7) protein expression in 20% of tumor samples. This expression was associated with a more favorable prognosis [53].

The analysis of immunoproteasome expression patterns in 9491 tumor samples from TCGA (33 cancer types), based on bulk RNA sequencing data, revealed that elevated immunoproteasome expression is associated with improved overall survival (OS) rates and enhanced responses to ICP blockade therapies in various cancers, including skin melanoma, NSCLC, breast cancer, bladder cancer, and thymic cancer. Tumors exhibiting high levels of immunoproteasome expression exhibited cytotoxic immune cell infiltration and upregulation of IFN-γ and tumor necrosis factor-alpha (TNF-α) pathways in tumor cells [54]. As these data are derived from bulk tumor transcriptomic analyses, the measured expression reflects contributions from both tumor cells and non-tumor components of the TME. The strong association with CD8+ T-cell infiltration and inflammatory cytokine pathways indicates that a substantial fraction of this signal likely arises from infiltrating immune cells. This interpretation is supported by studies using immunohistochemistry and molecular analyses showing that professional APCs within tumors express high levels of immunoproteasome subunits, whereas tumor cell expression can be heterogeneous and, in some cases, downregulated [43,51,54,55].

Other studies revealed that the dysregulation of the PSMB8 and PSMB9 immunoproteasome genes can alter the repertoire of peptides presented on MHC class I molecules, allowing tumors to evade immune detection [43,44]. These observations support the potential utility of immunoproteasome expression as a candidate biomarker of tumor immunogenicity and as a potential predictor of response to ICP blockade. However, their interpretation should consider the relative contribution of tumor versus immune cell expression. Most of these studies evaluated immunoproteasome expression in bulk tumor or tissue samples, correlating transcriptomic levels with intratumoral immune features such as CD8+ T-cell infiltration. Within the TME, immunoproteasome expression may arise from malignant cells; however, it often predominantly reflects expression by infiltrating immune cells, particularly DCs and macrophages. These APCs constitutively or inducibly express immunoproteasome subunits and play a central role in antigen processing and immune recognition [55].

The immunoproteasome contributes to both antitumor immunity and, in certain settings, tumor progression [56,57]. In some cancers, higher immunoproteasome expression enhances antigen processing and presentation, supporting immune recognition and correlating with improved OS, as observed in skin melanoma, NSCLC, breast, bladder, and thymic cancers. Conversely, in glioma, acute myeloid leukemia, oral, and renal cancers, elevated immunoproteasome expression is associated with poorer OS, indicating a potential pro-tumor effect. Therapeutic strategies targeting the immunoproteasome should therefore take these opposing roles into account, with activators potentially beneficial where expression favors survival and inhibitors considered where high expression is linked to worse outcomes [54]. These divergent associations likely reflect differences in the relative contribution of tumor cell expression and immune infiltration, as well as the overall immune context of the TME.

In cancers with worse OS rates, increased proteasome activity has been linked to the degradation of apoptosis-promoting factors, thereby allowing cancer cells to evade programmed cell death [58,59]. Accordingly, alterations in proteasome activity may influence tumor progression through both immune-dependent mechanisms (via antigen presentation) and immune-independent mechanisms (via regulation of cell survival pathways). The therapeutic efficacy of checkpoint inhibitors is often limited by the immunosuppressive TME and insufficient presentation of TAAs.

Selective immunoproteasome inhibition can broaden the antigenic peptide repertoire, enhancing T cell recognition of tumor cells. Combined with checkpoint blockade, it may also suppress immunosuppressive cytokine production and reduce PD-L1 expression on tumor and myeloid cells. Together, these effects promote the infiltration, activation, and cytotoxic function of tumor-infiltrating lymphocytes (TILs), resulting in a more potent and sustained anti-tumor immune response [60].

Overall, therapeutic approaches targeting the immunoproteasome should acknowledge its multifaceted potential roles. On the one hand, the immunoproteasome exerts an antitumor effect via its canonical contribution to antigen presentation in certain cancer types. On the other hand, it promotes tumor cell survival beyond antigen presentation functions in others.

##### ERAP

Another mechanism compromising APM function is related to ERAP, an IFN-γ-inducible aminopeptidase localized in the ER that trims precursors of antigenic peptides for MHC class I presentation. ERAP1 trims long peptides (9–16 residues) in the ER to the optimal 8–10 residue length required for MHC class I binding [61]. Although this activity is essential for generating antigenic peptides, ERAP1 can also overtrim or degrade certain 9-mer peptides, thereby reducing the pool of peptides available for MHC class I presentation [62]. Its activity is influenced by inflammatory signals; in the TME, IFN-γ produced by infiltrating immune cells upregulates ERAP1 expression, which in turn modulates MHC class I peptide generation [62]. Consequently, the repertoire of peptides presented by MHC class I reflects a balance between precursor availability and ERAP1 activity.

Genetic ERAP1 polymorphism has a strong effect on the epitope repertoire presented on MHC class I and can trigger an immune response by CD8+ T cells [63]. Analysis of naturally occurring ERAP1 haplotypes, defined by combinations of single-nucleotide polymorphisms (SNPs) across the gene in 20 individuals, revealed alleles with distinct enzymatic properties. Classified as normal, hypofunctional, or hyperfunctional, these variants affect peptide trimming for MHC class I presentation, shape the cell-surface antigenic peptide repertoire, and may influence an individual’s susceptibility to diseases [64].

Several studies highlighted the role of decreased ERAP1 levels as a mechanism for immune evasion by cancer cells, particularly in NSCLC [65,66] and CRC [67,68]. Wagner et al. conducted a study analyzing ERAP1 mRNA expression in tumors and adjacent non-tumor tissues from 61 NSCLC patients using real-time quantitative PCR (qPCR) [65]. Their findings demonstrated a significant reduction in ERAP1 mRNA levels in tumor tissues compared to non-tumor tissues [65]. The finding that the ERAP1 rs469783C allele, which correlates with increased mRNA expression levels, was associated with lower tumor-related mortality, providing support for a role of ERAP1 expression in anti-tumor responses [66]. In CRC, Gan et al. (2024) reported lower ERAP1 levels in tumors compared to normal tissues using bioinformatics and immunohistochemical analyses [67]. CRC patients with reduced ERAP1 expression exhibited poorer survival outcomes, and analysis of TCGA data showed that ERAP1 expression decreases in more advanced clinical stages [68], further supporting an association between lower ERAP1 levels and more aggressive disease. TCGA datasets are derived from bulk RNA sequencing. Therefore, the measured expression reflects the combined transcriptional contributions of tumor cells and non-tumor (immune, stromal) components within the TME. Although immunohistochemical data [67] support reduced ERAP1 protein expression within tumor cells, bulk transcriptomic associations cannot be attributed to malignant cells alone without complementary approaches such as single-cell analyses. Therefore, while reduced ERAP1 expression correlates with clinical outcomes, the signal may represent either altered expression within tumor cells, changes in the abundance of ERAP-expressing immune populations, or both. This distinction is particularly relevant when interpreting ERAP1 in the context of the TME, where inflammatory infiltrates and IFN-γ signaling can modulate its expression.

This downregulation in tumors may be attributed to microRNA-mediated silencing, particularly through the action of miR-223, which was shown to target and inhibit ERAP1 mRNA [65,69]. These findings collectively underscore the role of ERAP1 downregulation in promoting cancer immune evasion, suggesting its potential as a valuable biomarker for patient prognosis in NSCLC and CRC.

In a broader evaluation of ERAP1 and ERAP2 expression levels in 160 malignant non-lymphoid lesions, ERAP1 loss was reported in breast, ovary, liver, lung, colon and pancreas cancers [70]. In this study, it was suggested that malignant transformation causes ERAP1/ERAP2 losses, creating divergence in tumor-presented peptide repertoires in comparison to normal tissues [70].

Consistent with their roles in peptide trimming, loss of ERAP1 and ERAP2 is expected to reduce both the diversity and abundance of peptides available for MHC class I presentation. This allows cancer cells to evade immune detection by reducing both the diversity and abundance of antigens available for CD8+ T-cell recognition [71]. Clinically, such defects in antigen processing may influence responsiveness to T cell-based immunotherapies, including ICI, and could potentially be exploited therapeutically either by restoring antigen presentation pathways or by targeting ERAP activity to modulate the immunopeptidome.

ERAP1 has been shown to reduce the immunogenicity of melanoma cells by degrading a crucial tumor-specific antigenic peptide, the MART-126-35 epitope [72]. This epitope is produced by degradation of MART-1, a primary target for antigen-specific immunotherapy in melanoma patients. The degradation of the MART-1 epitope by ERAP1 downregulates its presentation, allowing the tumor to develop resistance against MART-1-targeted therapeutic interventions [72].

Additional studies further illustrate ERAP1′s impact on the immunopeptidome. Treatment of A375 melanoma cells with the potent ERAP1 inhibitor DG013A did not significantly change total MHC class I surface expression but substantially altered the repertoire of presented peptides [73]. DG013A caused the accumulation of peptides normally destroyed by ERAP1 and a reduction in peptides that require ERAP1-mediated trimming. Notably, around one-third of the affected peptides were predicted to bind strongly to MHC class I [73]. ERAP1 inhibition increased the cell-surface presentation of multiple MHC class I-optimized peptides [73], indicating that ERAP1 shapes the tumor immunopeptidome and influences the hierarchy of immunodominant epitopes recognized by cytotoxic T cells. Similarly, CRISPR-Cas9-mediated ERAP1 knockout in melanoma cell lines impaired generation of HLA-E peptides that engage the inhibitory receptor NKG2A, thereby enhancing tumor recognition and lysis by cytotoxic CD8+ T lymphocytes [74]. These observations reveal a context-dependent duality in ERAP function: whereas ERAP loss can promote immune evasion by reducing the peptide repertoire, it may also enhance CD8+ T-cell responses by unmasking higher-affinity or previously suppressed epitopes.

Altogether, these findings highlight the multifaceted role of ERAP1 in antigen processing. Its effects are shaped by factors such as allele type, expression level, peptide availability, and MHC class I context. It traditionally contributes positively to tumor immunogenicity via its canonical antigen processing role, but can also reduce tumor immunogenicity and immunopeptidome diversity by degrading tumor-specific antigenic peptides. Integrating bulk transcriptomic analyses with single-cell and protein-level approaches will be essential to clarify cellular sources of ERAP1 expression in tumors and to strengthen its translational application in cancer prognosis and therapy selection. Comprehensive evaluation of ERAP1 together with other components of the APM may also guide strategies aimed either at restoring impaired antigen processing or, in specific contexts, modulating ERAP1 activity to enhance anti-tumor immune recognition.

#### 2.3.2. Alteration of the Peptide Loading Complex Components

Following cytosolic peptide generation, a fraction of the produced peptides is transported into the ER by TAP. Peptides of appropriate length and sequence are subsequently loaded onto MHC class I molecules with the help of ER-resident chaperone proteins, including calreticulin, tapasin, and ERp57, forming the PLC.

##### Calreticulin

*Tumor-intrinsic role of calreticulin in antigen presentation*

The absence or dysfunction of calreticulin (CRT), a vital component of the PLC, significantly impairs peptide loading on MHC class I molecules in cancer cells [75]. In this context, CRT functions as a core chaperone within the PLC, directly influencing MHC class I assembly and stability. Using co-immunoprecipitation and flow cytometry, Arshad and Cresswell demonstrated that CRT mutations in myeloproliferative neoplasms reduce MHC class I surface expression by approximately 50%. These cancer-related CRT mutants fail to sustain normal PLC activity, leading to diminished MHC class I-antigen presentation and facilitating immune evasion by tumors that lose antigenicity [76].

The authors further illustrated that the delivery of MHC class I molecules to the cell surface is compromised due to MHC class I loading with suboptimal peptides, which destabilizes the peptide-MHC class I (pMHC-I) complex. Similar findings were observed upon expression of mutant calreticulin proteins with frameshift insertion and deletions (CRT-FSINS and CRT-FSDEL). In contrast, re-expression of wild-type calreticulin (CRT-WT) successfully restored MHC class I expression levels [76]. This study highlights a potential immune evasion mechanism employed by cancer cells to downregulate MHC class I expression, thus escaping immune detection and response. Immunohistochemical analysis has demonstrated significantly reduced levels of CRT in various cancers, including HNPCC sporadic right-sided tumors (RSTs) [77], bladder carcinomas (including urethral carcinomas) [78], and primary maxillary sinus squamous cell carcinoma (SCC) [79] lesions.

These findings underscore the tumor-intrinsic role of CRT in maintaining effective antigen processing and presentation through the MHC class I pathway.

*Calreticulin in immunogenic cell death and tumor immunogenicity*

Beyond these tumor-intrinsic effects, CRT also shapes anti-tumor immunity through mechanisms independent of its role in peptide loading and antigen presentation, notably by promoting tumor immunogenicity. This is achieved by CRT’s exposure as a DAMP on the cell surface during immunogenic cell death (ICD).

Under conditions of cellular stress, CRT can translocate from the ER to the cell surface, where it functions as an “eat-me” signal facilitating the recognition and engulfment of dying tumor cells by DCs and other phagocytes [80]. This process enhances antigen uptake and facilitates the priming of tumor-specific T-cell responses, thereby contributing to the immune activation associated with ICD. The immunostimulatory role of CRT was first demonstrated in studies showing that its surface exposure is required for the immune response elicited by certain chemotherapeutic agents [81], while subsequent work established that ER stress signaling is associated with CRT externalization during ICD [82]. Later investigations confirmed the relevance of CRT exposure across multiple malignancies, including breast, colorectal, and lung cancers, where ICD-associated markers correlate with enhanced immune infiltration and improved therapeutic responses [83]. Consistently, in primary and metastatic high-grade serous ovarian carcinoma (HGSC), tumors displaying elevated CRT exposure exhibit transcriptional signatures indicative of adaptive immune activation, including pathways linked to T-cell activation, Th1 polarization, immune cell recruitment, cytotoxicity, DC activation, and antigen processing [84]. In this context, ER stress responses within the TME promote the emission of DAMPs that enhance phagocytosis and antigen cross-presentation by DCs, thereby supporting effective anti-tumor immune priming. Collectively, these observations indicate that CRT contributes to anti-tumor immunity by enhancing tumor immunogenicity, with downstream effects on antigen cross-presentation via MHC class I. From a translational perspective, CRT exposure during ICD represents a mechanism that can be therapeutically exploited, even in tumors with impaired antigen presentation. Interventions targeting CRT-dependent pathways should be tailored according to whether the primary limitation is intrinsic APM dysfunction or insufficient tumor immunogenicity.

Restoring CRT levels, whether to correct intrinsic PLC defects or to enhance ICD-mediated immunogenicity, may improve tumor immune recognition and boost the efficacy of cancer therapies.

##### ERp57

ERp57 deficiency may impair peptide-MHC I complex formation and thereby contribute to cancer progression

ERp57 forms a disulfide-linked heterodimer with tapasin. This interaction is required for stable assembly of MHC class I molecules and efficient loading of high-affinity peptides in the ER [85,86]. Indeed, in the absence of ERp57, MHC class I loading complexes are prone to aggregation [87]. Studies show that ERp57-deficient complexes exhibit compromised stability, with the result that MHC class I molecules form aggregates in the ER rather than being properly assembled and transported to the cell surface. This aggregation is linked to altered protein turnover in the ER [87]. ERp57 deficiency reduces the presentation of antigenic peptides on the cell surface, which weakens CD8+ T-cell recognition and allows tumor cells to evade immune detection [88]. Cancer cells often exploit such deficiencies to escape immune surveillance, leading to increased tumor growth and metastasis [27]. For instance, histochemical analyses have shown a downregulation of ERp57 in urethral carcinoma compared to normal superficial urethral cells [78]. The downregulation of ERp57 was also correlated with reduced HLA class I expression in sporadic RST colorectal cancer [77]. In a comprehensive study involving immunohistochemical analysis of tissue microarrays from 164 gastric cancer patients, Leys et al. identified a notable downregulation of ERp57 in tumors. This downregulation was significantly associated with advanced stages of the disease, greater tumor invasion depth, and poorer prognosis. Conversely, higher levels of ERp57 were linked to improved survival [89], suggesting a potential role of ERp57 as a prognostic biomarker in cancer management.

##### The TAP Transporter

Another key component of the MHC class I antigen presentation pathway is the TAP transporter. Immunohistochemical analyses have demonstrated a decrease in TAP-1 and/or TAP-2 expression levels in 21% of primary breast carcinoma lesions [90] and downregulation of TAP-1 in 63% of RCC samples [91]. The decreased level of these proteins correlated with the tumor grading, since their downregulation was observed more frequently in high-grade tumors [90]. Studies on TAP-deficient lung carcinomas revealed increased MHC class I surface expression, enhanced antigen presentation, and improved immune responses, upon transfecting these cells with TAP-1 genes [92,93,94].

Other studies revealed that genetic and transcriptional alterations affecting the TAP1 and TAP2 genes, including loss-of-function mutations, genomic deletions, and downregulation, can result in decreased MHC class I-peptide surface expression, which impairs the immune system’s ability to recognize and eliminate tumor cells [42,43].

Others have highlighted TAP1′s broader implications, including its influence on the TME, immune infiltration, and tumor progression. TAP-1 expression was studied in the GEPIA2 (Gene Expression Profiling Interactive Analysis 2) and GENT2 (Gene Expression across Normal and Tumor tissues) databases and revealed increased TAP-1 mRNA levels in breast, liver, lung, and ovarian cancers, suggesting that the TAP protein paradoxically promotes tumorigenesis in these cancers [95]. In another study, TAP-1 upregulation was associated with worse prognostic outcome in ovarian cancer [96]. In clear cell renal cell carcinoma (ccRCC), the most common subtype of RCC, TAP1 mRNA and protein expression levels were higher in tumor tissues than in normal tissues, as documented in TCGA and Clinical Proteomic Tumor Analysis Consortium (CPTAC) databases. TAP1 expression was positively correlated with advanced cancer stages [97]. Experimental validation through Transwell and Scratch assays indicated that TAP1 promotes tumor migration and metastasis of ccRCC cells. Indeed, knock-down of TAP1 inhibited migration of ccRCC cell lines in vitro, while analysis of database single-cell transcriptomes indicated that increased TAP1 expression was positively associated with EMT, a process that enhances cancer cell invasiveness and metastasis [97].

To explain this observation, it has been suggested that TAP-1 contributes to multidrug resistance (MDR) in human cancer cells [98,99]. Increased expression of TAP-1 has also been associated with resistance to specific chemotherapeutic agents, such as Mitogen-activated protein kinase (MAPK) kinase (MEK) inhibitors in pancreatic cancer cells. In xenograft models, knockdown of TAP-1 resulted in decreased tumor growth and increased apoptosis when treated with MEK inhibitors. Conversely, overexpression of TAP-1 in sensitive PDAC cells conferred increased resistance to MEK inhibitors [100]. In this context, it is important to note that the primary transporters implicated in MDR are other well-characterized members of the ABC transporter family, including P-glycoprotein or the multidrug resistance protein 1 (MDR1), also known as ABCB1 (ATP-binding cassette sub-family B member 1); the multidrug resistance-associated protein 1 (MRP1), also known as ABCC1 (ATP-binding cassette sub-family C member 1); and breast cancer resistance protein (BCRP), also known as ABCG2 (ATP-binding cassette sub-family G member 2), which have been extensively studied and are essentially known to mediate the efflux of a wide variety of drugs, thereby reducing their intracellular concentrations and effectiveness [101]. While TAP1 appears as a potential target in this context, extensive validation is required to elucidate its precise mechanisms and validate its potential as a therapeutic strategy to overcome MDR in some settings.

In gene expression arrays, CRC tumors with low infiltration by lymphocytes generally showed a downregulated expression of TAP1. This downregulation has been specifically linked to immune evasion, characterized by a low expression of various immune cell-specific markers, including those marking T cells (CD3+), cytotoxic T cells (CD8+), regulatory T cells (Forkhead Box Protein 3: FOXP3+), T helper cells (T-box transcription factor TBX21: Tbet+), M1 macrophages (Nitric oxide synthase 2: NOS2+), and M2 macrophages (CD163+) cells, and was associated with poor prognosis in stage I-II-CRC patients [102]. Moreover, TAP1 expression was inversely correlated with DNA methylation at CpG sites located near the TAP1 promoter upstream of exon 1, suggesting that TAP1 methylation could be a putative mechanism for TAP1 downregulation. TAP1 expression is associated with immune cell infiltration within the TME. Therefore, its reactivation may represent a strategy to restore tumor-specific immune responses in CRC patients.

A preliminary study identified mutations in the TAP1 gene, specifically a frameshift mutation in the Buf1280 melanoma cell line, which was derived from a metastatic lesion of a melanoma patient [103]. This mutation resulted in the loss of a functional TAP1-TAP2 complex, leading to diminished peptide loading and reduced expression of MHC class I on the cell surface. A more recent study investigated the significance of TAP1 expression levels across various cancer types (pan-cancer), focusing on its potential as a prognostic biomarker and its role in the antitumor immune response [104]. TAP1 mRNA expression was significantly higher in most cancer tissues compared to normal tissues across 27 different cancer types. Notably, cancers such as cervical, endocervical, and cholangiocarcinoma exhibited particularly high levels of TAP1 mRNA. Western blot assays confirmed that TAP1 protein levels were also elevated in tumor samples compared to adjacent normal tissues, aligning with the RNA expression data. For instance, glioblastoma samples showed significantly higher TAP1 protein levels [104]. The analysis also included genetic data from TCGA, revealing that amplification, defined as an increase in the number of copies of a gene in a genome, was the most common alteration of the TAP1 gene across various cancers. However, the overall mutation frequency was relatively low, suggesting that while TAP1 is frequently overexpressed, TAP gene amplification may not play a substantial role in cancer progression. The study further demonstrated that elevated levels of TAP1 are associated with increased infiltration and activity of immune cells, particularly T-lymphocytes, as well as the presence of immunotherapeutic biomarkers indicative of effective immunotherapeutic responses [104].

TAP1 expression can be elevated in certain contexts. However, higher expression does not necessarily result in enhanced anti-tumor immunity. In certain cancers noted above, increased TAP1 has been linked to poor prognosis, tumor progression, metastasis, and drug resistance, highlighting its context-dependent role. Even when TAP1 expression is associated with high immune cell infiltration, therapeutic strategies targeting its expression require caution. Overexpression in tumors may reflect tumor-specific adaptations of the APM or interactions with the TME. It could be driven by immune infiltrates secreting IFN-γ or by the loss of physiological regulation of TAP gene expression as tumor cells dedifferentiate. Considering TAP’s potential MDR-like function, its overexpression might also enhance drug efflux and chemoresistance, offering a selective advantage to tumor cells. As a result, modulating TAP1 could have both beneficial and adverse effects on tumor progression, clinical outcomes, and anti-tumor immune responses, presenting a paradoxical scenario in which TAP overexpression may simultaneously reflect immune activation and tumor adaptation.

Another paradoxical finding is the emergence of cytosolic peptides known as TEIPP (T cell epitopes associated with impaired peptide processing) in tumor cells that are deficient in TAP [105,106]. Although TAP loss is expected to reduce conventional antigen presentation and limit CD8+ T-cell recognition, these TEIPP peptides are nonetheless processed and presented on MHC class I, representing tumor-specific neoantigens that are absent in healthy tissues and thus attractive targets for future immunotherapeutic strategies [107,108,109]. TEIPP antigens are discussed in greater detail in a subsequent section focusing on APM gene signatures and their relationship to antitumor immune responses and ICP therapy. However, these alternative and context-dependent mechanisms by which TAP influences tumor progression and therapeutic response remain incompletely understood, underscoring the complex role of APM components in cancer biology.

TAP1 can either enhance immune recognition through MHC class I antigen presentation or support tumor-intrinsic pathways that promote treatment resistance and tumor survival. This duality underscores the need for caution in therapeutic strategies targeting this pathway. Because these associations are largely derived from retrospective bulk and immunohistochemical data, causal relationships in human tumors remain incompletely defined. Strategies aimed at modulating TAP1 must therefore carefully balance its effects on antigen presentation against its potential impact on drug transport and tumor survival and should be evaluated in prospective studies that integrate both immune and clinical outcomes.

##### Tapasin

Tapasin (or TAPBP), is another key component of the APM, which promotes binding of peptide ligands providing sufficient stability to MHC class I molecules. Research has demonstrated that the absence of tapasin significantly reduces the diversity of MHC class I-presented peptides on the cell surface compared to wild-type cells [110]. Loss of tapasin impacts the selection of C-terminal amino acids in peptides processed for MHC class I presentation, resulting in weaker and less stable binding of these peptides to MHC class I molecules [110]. Lou et al. investigated the effects of human tapasin on murine lung carcinoma cells (CMT.64). The authors infected these cells with an adenovirus expressing human tapasin (AdhTpn), which led to enhanced MHC class I expression, improved immunogenicity, and more effective antigen presentation [111]. These results show the pivotal role of tapasin in optimizing the immune response through its influence on peptide-MHC class I interactions.

In primary maxillary sinus SCC lesions, immunohistochemical analysis demonstrated a reduced expression of tapasin in 69 out of 70 SCC samples [79]. This correlated with a downregulation of HLA class I levels. Notably, the levels of both tapasin and HLA class I expression were significantly associated with the infiltration of T cells into tumor lesions. Furthermore, tapasin expression showed a significant correlation with tumor differentiation. The degree of differentiation of tumor cells corresponds to their similarity to normal tissue and is scored based on histopathological criteria. Well-differentiated tumors closely resemble normal cells and have a better prognosis, moderately differentiated tumors show intermediate characteristics, and poorly differentiated tumors appear disorganized and are associated with a worse prognosis. Higher levels of tapasin were associated with better differentiation of tumor cells, while the downregulation of tapasin and HLA class I was linked to reduced patient survival [79], indicating that tapasin may influence the clinical progression of the disease. In a complementary study, immunohistochemical analysis revealed defective tapasin expression in 80% of RCC lesion samples [91]. Other studies have investigated APM genes, including TAPBP, across various tumor types, revealing differential effects on immune responses and treatment outcomes. For instance, stable cell surface MHC class I-peptide complexes depend on expression of TAPBP and other APM components, which in turn influence recognition of tumor cells by cytotoxic CD8+ T cells [42]. Collectively, these findings underscore the role of tapasin as a component of the APM, essential for effective antigen presentation, T cell activation, and robust anti-tumor immunity.

#### 2.3.3. Cancer-Associated Modulation of the MHC Class I Molecule Itself

Several studies have highlighted processes altering the function of MHC class I molecules. One mechanism involves the nitration and nitrosylation of the MHC class I peptide binding site, facilitated by peroxynitrite (PNT) produced by myeloid-derived suppressor cells (MDSCs) [112]. Research has shown that tumor-infiltrating MDSCs release reactive nitrogen species (RNS), including PNT, into the TME [113]. This release was shown to disrupt T-cell-mediated immune responses by altering the structure of the TCR through RNS production [114,115,116]. In a more recent study, Tcyganov et al. revealed that, besides directly nitrating the MHC peptide binding site thereby impairing peptide binding, PNT can modify peptides bound to MHC class I. Interestingly, some peptides exhibit greater sensitivity to PNT than others [117]. This modification can render tumor cells resistant to antigen-specific CTLs, underscoring the clinical significance of these mechanisms in tumor immunity.

Clancy-Thompson and collaborators investigated how the affinity of antigen binding to MHC class I influences the priming of CD8+ T cells and subsequent tumor suppression, with a specific focus on the melanoma antigen TRP1 (tyrosinase-related protein 1) [118]. To investigate the effects of binding affinity, a point mutation (K8) reported to destabilize the peptide interaction with the MHC binding groove was introduced in the MHC class I molecule. This mutation led to a marked decrease in the priming of CD8+ T cells [118], indicating that effective T cell priming is closely associated with the strength of the antigen-MHC class I interaction. Additionally, tumor growth was accelerated in the presence of this mutation, suggesting that altered binding affinity not only impacts T cell activation but also has direct consequences for tumor control.

In Pancreatic Ductal Adenocarcinoma (PDAC), tumor cells exploit autophagy as a mechanism to reduce MHC class I surface expression and evade immune recognition. Yamamoto et al. demonstrated that the autophagy cargo receptor NBR1 (neighbor of BRCA1 gene 1) mediates the sequestration of MHC class I molecules within autophagosomes and showed that pharmacological or genetic inhibition of autophagy increases MHC class I surface expression [119], supporting the concept that autophagy acts as a regulator of MHC class I cell surface availability.

Building on this mechanistic framework, Cheung et al. demonstrated that, in PDAC, MHC class I molecules undergo autophagy-dependent degradation and identified progranulin (PGRN), a multifunctional regulator of autophagy, as a key mediator of this process contributing to tumor immune evasion [120]. Their findings indicated that elevated levels of PGRN correlate with diminished MHC class I expression, which in turn is associated with reduced CD8+ T cell infiltration and cytotoxicity, as well as decreased survival rates in PDAC patients. Notably, when PGRN was inhibited through RNA interference, there was a marked increase in MHC class I surface expression in PDAC cells. This increase was linked to impaired clearance of autophagosomes under PGRN-deficient conditions, suggesting that PGRN participates in modulating immune evasion mechanisms in PDAC [120]. These observations were further validated in vivo using PDAC mouse models, which demonstrated positive outcomes following PGRN antibody-mediated blockade, including enhanced MHC class I surface expression and a significant delay in tumor initiation and progression [120].

In other contexts, it has been reported that dysregulation of Endoplasmic Reticulum-Associated Degradation (ERAD), a pathway that physiologically induces dislocation of misfolded/misassembled MHC class I molecules from the ER to the cytosol followed by their degradation, can result in compromised export of MHC class I molecules to the plasma membrane [2].

In another study, Shukla et al. conducted a comprehensive analysis of whole-exome sequencing (WES) data, identifying 298 non-silent mutations in HLA class I genes across tumors from 266 patients [121]. Their findings revealed that most of these mutations resulted in loss of function, leading to the production of non-functional MHC class I molecules. Notably, the highest mutation frequencies were observed in regions critical for MHC class I function, particularly in exon 4 (40%), which encodes the α3 domain of the HLA protein that interacts with the CD8 co-receptor on T cells [122,123]. Additionally, exons 2 and 3, encoding the α1 and α2 domains of the HLA molecule that form the peptide-binding pocket, accounted for 35% of the mutations. Mutations in these exons can disrupt the stability of the MHC-peptide complex, thereby impairing antigen presentation [121]. Furthermore, somatic mutations in HLA class I genes were found to be prevalent in various cancers, including colon adenocarcinoma, head and neck cancer, lung squamous cell carcinoma, and stomach cancer. This reinforces the idea that HLA mutations act as a common oncogenic mechanism and can be viewed as defects that may promote tumor progression and development by impairing the effectiveness of the MHC class I antigen presentation pathway. In an earlier study, Koopman et al. demonstrated the occurrence of mutations in HLA class I genes across various cervical cancer cell lines, which resulted in the formation of stop codons and consequently led to the loss of expression of MHC class I molecules [124]. They later identified genetic mutations in fresh cervical tumors with loss of heterozygosity (LOH) for HLA class I molecules. Altered HLA class I presentation was observed in 90% of the tumors analyzed, with some cases exhibiting a complete loss of antigen presentation, while others showed downregulation of MHC class I surface expression [125]. In other studies, Maleno et al. studied laryngeal carcinomas and detected HLA class I genetic changes associated with phenotypic alterations in 77% of cases [126]. Their results included HLA-I haplotype loss due to LOH in 36% of tumors, total loss of HLA class I expression in 11% of cases, allelic loss in 10% of tumors, and downregulation of MHC class I presentation in 20% of all cases. In the context of gastric cancers [127] and esophageal squamous cell carcinomas (ESCCs) [128,129], the LOH at the 6p21.3 locus was also associated with MHC class I downregulation. HLA class I LOH was also observed in colorectal tumors with high microsatellite instability (MSI-H) [77]. The latter are believed to face strong selective pressure to evade CTL activity since they produce large amounts of immunogenic peptides due to their genetic instability, which leads to frameshift mutations [130].

Altogether, these findings underline the key role of genomic mutations in downregulating MHC class I and disrupting antigen presentation. They highlight the clinical importance of further research in this area.

Other findings underscore the role of β2m (β2-microglobulin) in maintaining MHC class I stability and function, impacting subsequent immune responses. Research by Zhou et al. on head and neck squamous cell carcinoma (HNSCC) data sets in TCGA demonstrated that inhibiting EZH2 (Enhancer of Zeste Homolog 2) leads to increased MHC class I expression [131]. EZH2 is a histone-lysine N-methyltransferase that regulates gene expression by catalyzing the addition of methyl groups to lysine 27 of histone H3 (H3K27me3). The authors proposed that EZH2 suppresses antigen presentation by downregulating β2m expression, as its inhibition reduces the H3K27me3 modification on the β2m promoter [131]. In a study involving immunohistochemical analysis of 75 tumors from patients with Hereditary Nonpolyposis Colorectal Cancer (HNPCC), it was revealed that loss of β2m expression correlates with a loss of HLA class I expression in 46.9% of cases negative for HLA class I [77]. Similarly, another study found reduced β2m levels on the membranes of urethral carcinoma cells compared to normal superficial urothelium cells [78].

Moreover, breast cancers with low MHC class I expression (MHC I-low) have been found to contain fewer TILs compared to their MHC I-high counterparts [132]. This suggests that reduced MHC class I expression may contribute to immune evasion by limiting the recruitment of anti-tumor T cells. Furthermore, the loss or downregulation of MHC class I has been associated with worse clinical outcomes in various cancers, including melanoma, glioblastoma, colorectal cancer (CRC), bladder cancer, uterine cancer, cervical cancer, head and neck cancers, and breast cancer [133,134,135]. This underscores the crucial role of MHC class I expression in effective anti-tumor immune responses and the contribution of low expression to tumor progression and metastasis.

Most evidence on HLA LOH and B2M alterations derives from bulk exome sequencing, which may underestimate the spatial heterogeneity of HLA loss within tumors. Prospective studies that integrate single-cell genomics with comprehensive immune profiling are therefore needed to clarify the causal role of HLA and B2M alterations and to define their implications for immunotherapy.

#### 2.3.4. Transcriptional and Epigenetic Control of APM Components

Having outlined the APM components involved in peptide production, loading, and presentation, it is important to consider the epigenetic and transcriptional regulators that govern the expression of these elements. Such regulators have emerged as potential targets for immunotherapeutic intervention, and their expression profiles may also provide valuable prognostic insight. This section focuses on key transcriptional regulators implicated in APM regulation, namely NOD-like receptor C5 (NLRC5), Polycomb Repressive Complex 2 (PRC2), and Interferon Regulatory Factor 2 (IRF-2).

NLRC5 (also known as CITA, MHC class I transactivator) is an IFN-γ-inducible transcriptional regulator whose loss reduces the expression of MHC class I and multiple APM genes [2,136,137].

It has been demonstrated that the levels of MHC class I are closely correlated with the expression of NLRC5 [138] and are mediated by an enhanceosome, a specialized protein complex that forms at enhancer regions of DNA, playing a crucial role in the regulation of gene expression [139]. In line with this, cells deficient in the enhanceosome component RFX5 display a defective MHC class I expression phenotype similar to that observed in NLRC5-deficient cells [139]. This finding underscores the critical role of the enhanceosome in regulating NLRC5 function and, consequently, the expression of MHC class I-related genes. Recent studies have uncovered that in various cancers, the Polycomb Repressive Complex 2 (PRC2) silences NLRC5. Inhibition of PRC2 leads to the derepression of NLRC5, resulting in increased MHC class I expression [140].

To further investigate this mechanism, a genome-wide CRISPR-Cas9 screen was conducted using the MHC class I-low K-562 erythroleukemia cell line [140]. The screening revealed that PRC2 actively silences the genes encoding MHC class I heavy chains. This silencing is believed to occur through the H3K27me3 histone methylase. Notably, when the EZH2 methyltransferase subunit of PRC2 is inhibited, MHC class I levels are restored, and H3K27me3 levels are significantly reduced [140]. This observation highlights another mechanism by which PRC2 can regulate the expression of MHC class I-related genes, suggesting potential therapeutic avenues for enhancing immune recognition in cancer.

In a separate study, NLRC5 was identified as a significant target for immune evasion across various cancer types [141]. The expression and function of NLRC5 are compromised through mechanisms such as methylation of CpG sites in the NLRC5 promoter, as well as mutations (predominantly loss-of-function mutations) and copy number losses in the NLRC5 gene [141]. The researchers corroborated these findings with multiple tumor types, demonstrating that downregulation of NLRC5 is associated with reduced expression of MHC class I-related genes, decreased CD8+ T-cell cytotoxicity, and poor patient prognosis and survival outcomes [141].

It is important to note, however, that NLRC5 has been linked to tumor-promoting functions. For instance, MHC class I-positive and nuclear NLRC5-positive stage III NSCLC patients were reported to have shorter OS rates [142]. High levels of NLRC5 were also found in hepatocellular carcinoma (HCC). In this context, NLRC5 overexpression was shown to enhance tumor cell proliferation, migration, and invasion [143]. The authors further related the negative effects of NLRC5 in HCC to its role in coordinating the activation of Wnt/β-catenin signaling, a pathway that was reported to activate various target genes, such as C-myc, cyclinD1, metalloproteinases, or VEGF, involved in cell proliferation, adhesion, migration, and tumorigenesis [143]. Thus, NLRC5 has a dual role in cancer: it can boost immune responses via MHC class I induction, yet may also promote tumor progression.

Another epigenetic modulator associated with antigen presentation is IRF-2. Kriegsman et al. demonstrated that the transcriptional regulator IRF2 plays an important role in tumor immune evasion [144]. The authors found that IRF2 is frequently downregulated in various primary human cancers, including lung, colon, breast, and prostate cancers [144]. They established that IRF2 functions as a positive regulator of several components involved in MHC class I antigen presentation, such as immunoproteasomes, TAP, and ERAP1. The downregulation of IRF2 was shown to reduce transport of peptides from the cytosol to the ER and impair N-terminal peptide trimming, which negatively impacts MHC class I surface presentation [144]. Furthermore, their findings indicated a correlation between reduced IRF2 levels and increased expression of PD-L1, an ICP protein that inhibits CD8+ T cell activity against tumors and that is normally repressed by IRF2 [144]. Importantly, the loss of antigen presentation due to IRF2 downregulation can be reversed through the interferon-stimulated induction of IRF1, another transcription factor [144].

Overall, these findings demonstrate that the loss of IRF2 not only impairs antigen presentation but also facilitates immune evasion through increased PD-L1 expression. They also highlight the possibility of developing therapeutic approaches focused on either restoring the function of IRF2 or boosting the activity of IRF1. Such strategies could enhance MHC class I antigen presentation, ultimately strengthening the immune response against tumors.

NLRC5, PRC2, and IRF2 establish a regulatory network that orchestrates transcriptional and epigenetic control of antigen presentation and tumor immune evasion. Within this network, (i) NLRC5 and PRC2 regulate MHC class I surface expression through transcriptional activation and epigenetic silencing, respectively; (ii) IRF2 determines the quality of the peptide repertoire by controlling the expression of immunoproteasomes, TAP, and ERAP1; and (iii) IRF2 also modulates checkpoint ligand expression, including PD-L1, thereby facilitating immune evasion. By orchestrating both antigen display and inhibitory signaling, these regulators collectively shape the ability of CD8+ T cells to recognize and eliminate tumor cells, highlighting their potential as actionable targets for immunotherapeutic intervention.

**Table 1:** **APM components in MHC class I pathway: Dysregulation, mechanisms, and cancer associations.** This table classifies antigen presentation machinery (APM) components by their roles in MHC class I antigen presentation. It details dysregulation states (e.g., downregulation, mutations), resulting effects on immune recognition, underlying mechanisms, detection levels/techniques, associated cancer types, and key references.

Role in APM	APM Component	Alteration Type	Alteration-to-Effect Context	Evidence Level (Technique)	Clinical Association (Prognosis/ICI Response)	Cancer Type	References
Peptide generation in MHC class I presentation pathway	Immunoproteasome (LMP2/PSMB9, LMP7/PSMB8, LMP10/PSMB10)	Post-translational alteration (downregulation)	Loss of immunoproteasome subunits impairs proteasomal cleavage of tumor antigens, leading to a reduced antigenic peptide generation for MHC class I presentation	DNA-level (genomic profiling), protein-level (IHC)	-Worse prognosis associated with lower expression - Higher expression associated with better response to ICI therapy, taking into account the tumor-specific immune infiltrate pattern	RCC, HNSCC, NSCLC	[50,51,52,53,54]
	ERAP1	Genetic alteration (downregulation)	Decreased ERAP1 limits antigenic peptide supply	DNA-level (qPCR), protein-level (IHC)	mRNA expression levels not associated with survival and prognosis	NSCLC, CRC, Ovary	[62,64,65,67,68,70]
		Genetic alteration (variants classified as hypo-/hyper-active)	Certain variants degrade epitopes excessively which reduces peptide trimming and alters peptide repertoire	DNA-level (bioinformatics)	Poor prognosis associated with the number of unfavorable genotypes	NSCLC	[66]
		Post-transcriptional alteration (downregulation)	Overexpressed miRNAs that silence ERAP1 genes lead to reduced and altered peptide repertoire	DNA-level (bioinformatics, miRNA inhibition)		Breast	[69]
		Post-translational alteration (inhibition)	Induced inhibition of ERAP1 through a potent inhibitor (DG013A) causes a shift in the immunopeptidome and epitopes expressed on the surface of MHC class I molecule	Protein-level (Liquid chromatography–tandem mass spectrometry)		Melanoma	[73]
	ERAP2	Genetic alteration (downregulation)	Loss affects cooperation with ERAP1 in peptide trimming, altering peptide repertoire balance in ER	Protein-level (IHC)		Breast, Liver, Colon, Pancreas cancers	[70]
Modulation of peptide-loading complex (PLC) components	Calreticulin (CRT)	Genetic alteration (mutated CRT)	CRT mutations prevent normal PLC formation, leading to disruption of PLC function and downregulated MHC class I surface expression	Functional-level (molecular analysis)	- Mutated CRT expression associated with poor prognosis - Better prognosis observed with wild-type CRT	Myeloproliferative neoplasms,	[76]
		Undefined alteration (downregulation)	CRT downregulation led to a reduced expression of MHC class I aiding in tumors escaping immunosurveillance	Protein-level (IHC)	Poor prognosis associated with lower expression of CRT	CRC, Bladder, HGSC	[78,79,80]
	ERp57 (PDIA3)	Genetic alteration (downregulation)	Loss/downregulation causes MHC protein aggregation in ER and misfolding within PLC causing instability of MHC class I-peptide complexes and impaired antigen export	Protein-level (IHC), functional-level (Fluorescence resonance energy transfer analysis)	Better prognosis associated with higher expression	Gastric adenocarcinoma, Urethral carcinoma	[78,87,89]
	TAP1	Epigenetic or genetic alteration (downregulation)	Defects block peptide translocation to the ER, impairing peptide transport and altering MHC class I presentation	DNA-level (bioinformatics), RNA-level (RT-PCR), protein-level (Western blot)	Better prognosis correlated with higher expression	Melanoma, RCC, CRC, NSCLC, SCLC, BRCA, liver cancer	[90,91,92,93,94,95,102,103]
		Epigenetic or genetic alterations (overexpression)	TAP1 overexpression promoting EMT or drug resistance among tumor cells and involved in emergence of neoantigens	DNA-level (bioinformatics), RNA-level (qRT-PCR), protein-level (IHC), functional-level (siRNA inhibition)	Worse prognosis associated with overexpression	Ovarian cancer, ccRCC, PDAC,	[96,97,98,99,100,101]
	TAP2	Post-translational alteration (downregulation)	Loss of function disrupts ER-peptide loading for antigen presentation, causing decreased peptide import and MHC class I expression	DNA-level (genomic profiling), RNA-level (RT-PCR), protein-level (IHC)	Improved survival correlated with higher expression	Breast carcinoma, RCC, HNSCC	[51,90,91]
	Tapasin (TAPBP)	Undefined alteration (downregulation)	Loss impairs peptide optimization within the PLC and weakens antigen presentation, causing reduced MHC class I-peptide loading and surface stability	RNA-level (RT-PCR), protein-level (IHC)	Reduced survival correlated to lower expression	RCC, Maxillary sinus SCC	[79,91]
Cancer-associated modulation of MHC class I	β2-Microglobulin (B2M)	Epigenetic inhibition (downregulation)	EZH2-mediated H3K27me3 methylation suppresses B2M transcription leading to loss of MHC class I complex stability and surface expression	DNA-level (CRISPR-Cas9), RNA-level (qRT-PCR), protein-level (ELISA), functional-level (flow cytometry)	- Poor prognosis correlated with overexpression of EZH2. - Using combined immunotherapy with EZH2 inhibition to overcome ICI-resistant tumors.	HNSCC	[131]
		Undefined alteration (downregulation)	B2M deficiencies associated with lower expression of MHC class I and surface antigens	DNA-level (bioinformatics), protein-level (IHC)		HNPCC, Urothelial carcinoma	[77,78]
	MHC class I heavy chains (HLA-A/B/C)	Genetic alteration (downregulation)	Mutations and loss of heterozygosity (LOH) disrupt peptide binding and stability of the MHC class I complex, causing loss of antigen presentation and immune evasion	DNA-level (Whole-exome sequencing, bioinformatics, PCR-LOH analysis), protein-level (IHC)	Poor survival correlated with lower expression	Colon adenocarcinoma, SCLC, Head and Neck, Gastric, glioblastoma, ovarian cancer, laryngeal carcinoma, Cervical cancer	[77,121,122,123,124,125,126,127,128,129]
Transcriptional and epigenetic control of APM components	RFX5	Knockout cells deficient in RFX5	Loss of enhanceosome component mimics NLRC5 deficiency, leading to reduced MHC class I transcription and expression	Functional-level (functional genomic analysis)			[139]
	NLRC5	Epigenetic silencing (downregulation)	Silencing via PRC2-mediated H3K27me3 or CpG promoter methylation leads to downregulation and reduced transcription of MHC class I and APM genes	DNA-level (CRISPR-Cas9), protein-level (methylation and expression assays)	Poor prognosis correlated with lower expression	Colon, melanoma	[136,137,138,139,140,141]
		Undefined alteration (overexpression)	Overexpression promotes proliferation, invasion and migration of tumor cells	DNA-level (bioinformatics, qPCR), protein-level (IHC, Western blot)	Poor prognosis associated with higher expression	NSCLC, HCC	[142,143]
	IRF2	Undefined alteration (downregulation)	Loss reduces TAP and ERAP transcription while enhancing ICP expression, suppressing antigen presentation and increasing PD-L1 expression	DNA-level (CRISPR-Cas9), functional-level (transcriptomic and proteomic analysis)		Lung, Colon, Breast, Prostate cancers	[144]

### 2.4. APM Gene Signatures and Antitumor Responses in Immunotherapy

Because of their essential roles in antigen processing and presentation, the expression levels of APM genes can profoundly impact the immune landscape of tumors and the effectiveness of immunotherapeutic strategies. The advancement of immunotherapies for cancer represents a transformative approach to enhancing the immune response in patients. Key strategies include the use of ICI, cancer vaccines, or adoptive T cell therapies, which act through distinct mechanisms to disrupt the immunosuppressive signals within the TME and allow T cells to effectively recognize and destroy cancer cells [28,29,145]. Because these strategies ultimately depend on tumor antigen recognition by T cells, their efficacy is closely linked to the functional integrity of the APM: ICP blockade requires effective MHC class I-mediated presentation to sustain CD8+ T cell responses, whereas approaches such as TEIPP-targeting vaccines can exploit alternative peptide repertoires that emerge in the context of APM deficiencies; conversely, impaired antigen processing may limit the effectiveness of T cell-based therapies. Thus, alterations in APM components may differentially affect response depending on the class of immunotherapy and the underlying mechanism of antigen recognition.

In this section, we discuss some studies examining the relationship between APM components, immunotherapeutic responses in cancer, and APM gene signatures associated with antitumor outcomes.

For instance, as outlined in the previous discussion of TAP, TEIPPs constitute a distinct class of cytosolic peptides generated in tumor cells lacking functional TAP. TEIPPs are non-mutated self-antigens derived from housekeeping proteins that are presented by MHC class I exclusively on TAP-deficient cancer cells. When cancer cells downregulate the peptide pump TAP, presentation of TEIPP antigens is observed. TAP downregulation or deficiency induces a substantial reduction in the presentation of TAP-dependent peptides. The resulting loss creates an environment favoring the presentation of TEIPP antigens without competition from the immunogenic TAP-dependent peptides. TEIPP antigens can be recognized by CD8+ T cells to eliminate tumors that have escaped CTL responses [105,106]. CD8+ T cells specific for these TEIPPs undergo positive but little or no negative selection in the thymus and persist as naïve T cells, ready for activation; they do not cause autoimmunity as TEIPPs are not expressed by healthy tissue cells [107,108,109].

In 2018, a study aimed to identify potential HLA-A*02:01 epitopes in the absence of TAP protein in tumor cells [146]. A screening approach based on the whole normal human proteome was chosen as a starting point, since TEIPP antigens are non-mutated self-antigens. This approach yielded a total of 65 candidates, 40 of which bound to HLA-A*02:01, and 16 peptides were recognized by cognate CD8+ T cells. Of these 16 TEIPP peptides, some were tissue-restricted, but one of them, LRPAP1_21–30_, derived from the LDL receptor related protein associated protein 1 (LRPAP1), was of interest. This antigen is ubiquitously expressed and was therefore considered a universal HLA-presented tumor antigen in cancers with impaired antigen processing. CD8+ T cells specific for this epitope are able to recognize TAP-deficient, HLA-I^low^ lymphomas, melanomas, and renal and colon carcinomas, but not their healthy tissue counterparts [146]. This research highlights the therapeutic potential of targeting TEIPPs in cancer immunotherapy, emphasizing their unique neoantigen features and their relevance for treating tumors that have developed resistance to conventional therapies.

To further highlight the influence of TAP on immunotherapeutic outcomes, in 2019, Garrido et al. introduced a method that uses a chemically synthesized nucleolin aptamer conjugated with TAP small interfering RNA (siRNA) to transiently downregulate TAP expression in both human and murine tumor models [147]. The primary objective of this study was to address the challenge of tumor cells evading checkpoint and other immunotherapies. The use of the nucleolin aptamer-TAP siRNA conjugate successfully inhibited tumor growth across various models without demonstrating significant toxicity [147]. By inducing the expression of new antigens associated with TAP downregulation in tumor cells, this method enhanced the antitumor effects of ICP therapies, including PD-1 (Programmed Cell Death 1) antibodies, and facilitated the presentation of TAP-independent peptides [147].

Another study proposed a method to measure a tumor immunogenicity score (TIGS), which combines tumor mutational burden (TMB) and APM [148]. “APM signature” refers to a defined set of genes involved in antigen processing and presentation, including proteasome components (PSMB5-10), peptide transporters (TAP1, TAP2), peptide trimming enzymes (ERAP1, ERAP2), chaperones (CANX, CRT, PDIA3), TAPBP, B2M, and HLA class I genes (HLA-A, HLA-B, HLA-C), whose mRNA expression levels are used to generate a score reflecting antigen presentation capacity at the transcript level [148]. Analysis of the APM-gene signature score (comprising PSMB5, PSMB6, PSMB7, PSMB8, PSMB9, PSMB10, TAP1, TAP2, ERAP1, ERAP2, CANX, CRT, PDIA3, TAPBP, B2M, HLA-A, HLA-B and HLA-C) revealed significant variations in antigen presentation efficiency across 32 cancer types from the TCGA (The Cancer Genome Atlas Program) and indicated that it had an impact on the response rates to immunotherapies. For instance, breast cancer and prostate cancer typically exhibit high TMB and low ICI response rates, possibly linked to low APM scores. In contrast, ccRCC tends to have a good ICI response rate, possibly due to high APM scores [148]. The authors also showed that, in most TCGA cancer types with high APM scores, interferon alpha/gamma response gene signatures were enriched [148]. Given that IFN-γ has been reported to regulate APM gene expression in tumors [149], this result suggests that high APM scores are associated with induction of the interferon alpha/gamma signaling pathway. Therefore, stimulating interferon signaling in patients with cancer types that have low APM scores, such as prostate cancer and breast cancer, could be an interesting approach to enhance the response to immunotherapy (ICI).

In 2020, a study by Thompson and colleagues identified a gene signature linked to the APM and its impact on the response to ICP blockade therapy. The gene expression profiles of APM components were analyzed in metastatic chemotherapy-refractory NSCLC patients, as well as in two cohorts of advanced melanoma patients undergoing treatment with anti-PD1 or anti-PD-L1 (Programmed Cell Death Ligand 1) antibodies. A refined antigen processing signature was generated by removing genes that were not differentially expressed between responders and non-responders and showing a positive response to immune check-point blockade. This resulted in a score based on eight key genes associated with APM: B2M, CRT, NLRC5 (NOD-like Receptor C5), PSMB9, PSME1 (Proteasome activator complex subunit 1), PSME3 (Proteasome activator complex subunit 3), RFX5 (Regulatory Factor X5), and HSP90AB1 (heat shock protein 90 alpha family class B member 1). A higher APM score established in this manner was found to correlate strongly with improved therapeutic response, enhanced progression-free survival (PFS), and increased OS rates in these patient cohorts [44]. Importantly, these associations are predictive, derived from pre-treatment tumor biopsies, and indicate likely response to therapy rather than overall prognosis [44,148].

Interestingly, several well-known APM genes, such as TAP1/TAP2, MHC class I, tapasin, and ERAP1, were excluded because the signature focuses on genes whose expression correlates with clinical benefit from ICP blockade, rather than all components required for antigen processing. The selected genes reflect expression profiles associated with therapeutic benefit, whereas the excluded genes are constitutively expressed or regulated post-transcriptionally. Consequently, their mRNA abundance is not a reliable indicator of tumor immunogenicity or clinical response. By focusing on these eight genes, the signature links tumor-specific variation in APM transcription to clinical outcomes, underscoring the importance of antigen processing and presentation in immune-mediated cancer cell elimination.

A study by Şenbabaoğlu et al. in 2016 employed a gene expression-based computational method to profile the infiltration levels of 24 immune cell populations in 19 cancer types [150]. The study identified specific messenger ribonucleic acid (mRNA) signatures that were associated with patient prognosis. Indeed, elevated levels of CD8+ T cells, plasmacytoid DCs (pDC), T cells, cytotoxic cells, Natural killer (NK) cells, and neutrophils and low levels of Th2 and Treg cells were highlighted in ccRCC tumors compared to 18 other studied cancer types [150]. Moreover, a seven-gene APM signature that included MHC class I genes (HLA-A/B/C, B2M) and key genes responsible for antigen processing and loading (TAP1, TAP2, and TAPBP) was highly positively correlated with the immunogenicity of ccRCC tumors [150]. Unlike the predictive signatures described above, these observations are prognostic, reflecting overall patient outcome and immune context rather than response to a specific therapy. In contrast to Thompson’s study [44], this ccRCC APM signature shows that transcription factors such as NLRC5 and RFX5 maintain stable transcript levels and are not predictors of tumor immunogenicity. Instead, tumor immunogenicity in ccRCC appears to be shaped by variation in the expression of other canonical antigen-processing and presentation genes, suggesting that APM signatures vary depending on the cancer type.

Additionally, higher levels of Th17 cells and a favorable CD8+ T/Treg ratio were associated with improved survival outcomes. Conversely, high levels of Th2 cells and Tregs correlated with poorer prognosis. High Th2 and Treg cell levels showed a positive correlation with mutation load [150]. These correlations could be indicative of an immunosuppressive environment enriched in Treg and/or Th2 cells where tumors have escaped elimination by the immune system despite bearing a large number of potentially immunogenic mutations. RNA-Seq profiles of six ccRCC patients treated with the checkpoint inhibitor nivolumab (anti-PD1) were also analyzed in this study [150]. The patients who responded to the treatment showed higher T cell infiltration, APM, IFNG (interferon-gamma), and GZMB (granzyme B) levels, with the highest levels observed in the patient with a complete response. Thus, in ccRCC, a highly expressed APM promotes a more efficient anti-tumor response, showcasing its potential as a valuable biomarker for predicting treatment outcomes in ccRCC patients.

Overall, these results highlight the clinical relevance of understanding the role of MHC class I antigen presentation in cancer and the potential for targeting its components as part of personalized cancer treatment strategies. Implementation of personalized immunotherapy should integrate pre-treatment predictive APM signatures for ICP blockade response, consider alternative antigen presentation pathways for vaccine strategies, and account for prognostic APM signatures reflecting tumor immune context to support patient stratification and therapy selection.

## 3. Conclusions and Perspectives

Antigen presentation is a fundamental component of adaptive immunity and is indispensable for effective T-cell-mediated immune responses (Figure 2). The correct function of the MHC class I APM strongly influences tumor immunogenicity, immunoediting, and responses to therapy. Disruptions in peptide generation and loading, MHC class I stability or surface expression can profoundly alter the tumor immunopeptidome and represent a major route by which cancer cells avoid recognition by CD8+ T cells.

A key insight from the study of APM defects is that tumor immunogenicity is not fixed but changes over time. Tumors evolve under immune pressure and acquire genetic, epigenetic, and post-translational changes in APM components that promote immune escape. Alterations in immunoproteasome subunits, ERAP1/2, TAP, tapasin, calreticulin, ERp57, or MHC class I molecules themselves are often associated with reduced immune infiltration, worse clinical outcomes, and resistance to immunotherapy. However, their impact is context-dependent: in some settings, increased expression can support tumor progression, reshape immune responses, or limit therapeutic efficacy. This highlights the importance of interpreting APM alterations in relation to the tumor’s cellular state and the immune landscape of the TME.

Therefore, analysis of APM defects demonstrates that not all alterations have equal influence on tumor immunogenicity or disease progression. Assessing these defects according to their frequency, functional impact, and effect on immune recognition allows identification of those with the greatest effect on tumor visibility to T cells. This framework distinguishes components required for effective T-cell recognition from those driving immune evasion, linking mechanistic understanding to functional consequences.

Nevertheless, a major limitation in the field is that current assessments of APM defects often fail to capture the full complexity of tumor antigen presentation. APM dysfunction is often inferred from genomic or transcriptomic data, yet these measures do not always predict whether antigen processing or presentation is impaired at the protein level, nor do they account for post-translational regulation. Experimental functional assays are technically challenging and have been applied to only a limited set of tumors, which reduces the ability to generalize mechanistic conclusions. Tumor heterogeneity further complicates interpretation, as different regions within the same tumor can vary in their APM status. Some alterations, such as epigenetic downregulation of TAP, tapasin, or MHC class I, are reversible and potentially addressable through targeted interventions, whereas genetic deletions or truncating mutations in key APM genes are irreversible. Integrating analyses of the temporal dynamics of APM alterations, single-cell analyses, and spatial profiling of APM variation across tumor regions will be essential to distinguish therapeutically actionable defects from permanent ones, thereby guiding rational immunotherapy design and personalized treatment strategies.

Defective antigen presentation should therefore be viewed not only as a limitation but also as a therapeutic opportunity. Accordingly, understanding the mechanisms underlying APM dysfunction can guide the development of strategies to restore or modify antigen presentation. Personalized cancer vaccines based on tumor antigenic profiles and patient-specific immunity, adoptive T-cell therapies, and ICI targeting pathways such as PD-1 and CTLA-4 all depend, directly or indirectly, on effective antigen presentation to achieve lasting clinical benefit.

Additional approaches, including immune adjuvants, cytokine modulation, epigenetic therapies, targeting of proteasome or ERAP activity, and inhibition of autophagy-driven MHC class I degradation, provide further options to enhance tumor immunogenicity and convert immunologically “cold” tumors into “hot” tumors.

## Data Availability

No new data were created or analyzed in this study.

## Abbreviations

APC	antigen-presenting cell
APM	Antigen presentation machinery
BRCA1	Breast cancer type 1 susceptibility protein
CRT	calreticulin
ccRCC	clear cell renal cell carcinoma
CLIP	class II-associated invariant chain peptide
CRC	colorectal cancer
CTL	cytotoxic T lymphocyte
DAMP	Danger-Associated Molecular Pattern
EMT	Epithelial–Mesenchymal Transition
ER	Endoplasmic reticulum
ERAP	endoplasmic reticulum aminopeptidase protein
EZH2	Enhancer of Zeste Homolog 2
FOXP3	Forkhead Box Protein 3
HCC	hepatocellular carcinoma
HGSC	High-grade serous carcinoma
HLA	human leukocyte antigen
HNPCC	Hereditary Nonpolyposis Colorectal Cancer
HNSCC	head and neck squamous cell carcinoma
ICI	immune checkpoint inhibitor
ICP	immune checkpoint
IFN-γ	interferon-gamma
IRF	Interferon Regulatory Factor
LOH	loss of heterozygosity
MDSC	myeloid-derived suppressor cell
MHC	major histocompatibility complex
mRNA	messenger ribonucleic acid
NBR1	Neighbor of BRCA1 gene 1
NLRC5	NOD-like Receptor C5
NOS2	Nitric oxide synthase 2
NSCLC	Non-small cell lung cancer
OS	Overall survival
PAMP	Pathogen- Associated Molecular Pattern
PD-L1	Programmed Cell Death Ligand 1
PLC	peptide-loading complex
PRC2	Polycomb Repressive Complex 2
RFX5	Regulatory Factor X5
RNS	Reactive nitrogen species
SCC	squamous cell carcinoma
SLO	secondary lymphoid organs
TAA	Tumor-associated antigens
TAP	transporter associated with antigen processing
Tbet	T-box transcription factor TBX21
TCR	T-cell receptor
TEIPP	T cell epitopes associated with impaired peptide processing
TME	Tumor microenvironment
TNF-α	tumor necrosis factor-alpha
TRP1	Tyrosinase-related protein 1
TSA	Tumor-specific antigens
β_2_m	β_2_-microglobulin
